# Supplementary material for: Effects of Rho-Associated Kinase (Rock) Inhibitors (Alternative to Y-27632) on Primary Human Corneal Endothelial Cells
Source: Cells. 2023 May 3;12(9):1307. doi: 10.3390/cells12091307 (PMC10177577; doi:10.3390/cells12091307)
Supplement: Supplementary file 1 [file cells-12-01307-s001.zip › cells-2169359-supplementary.pdf]

cells

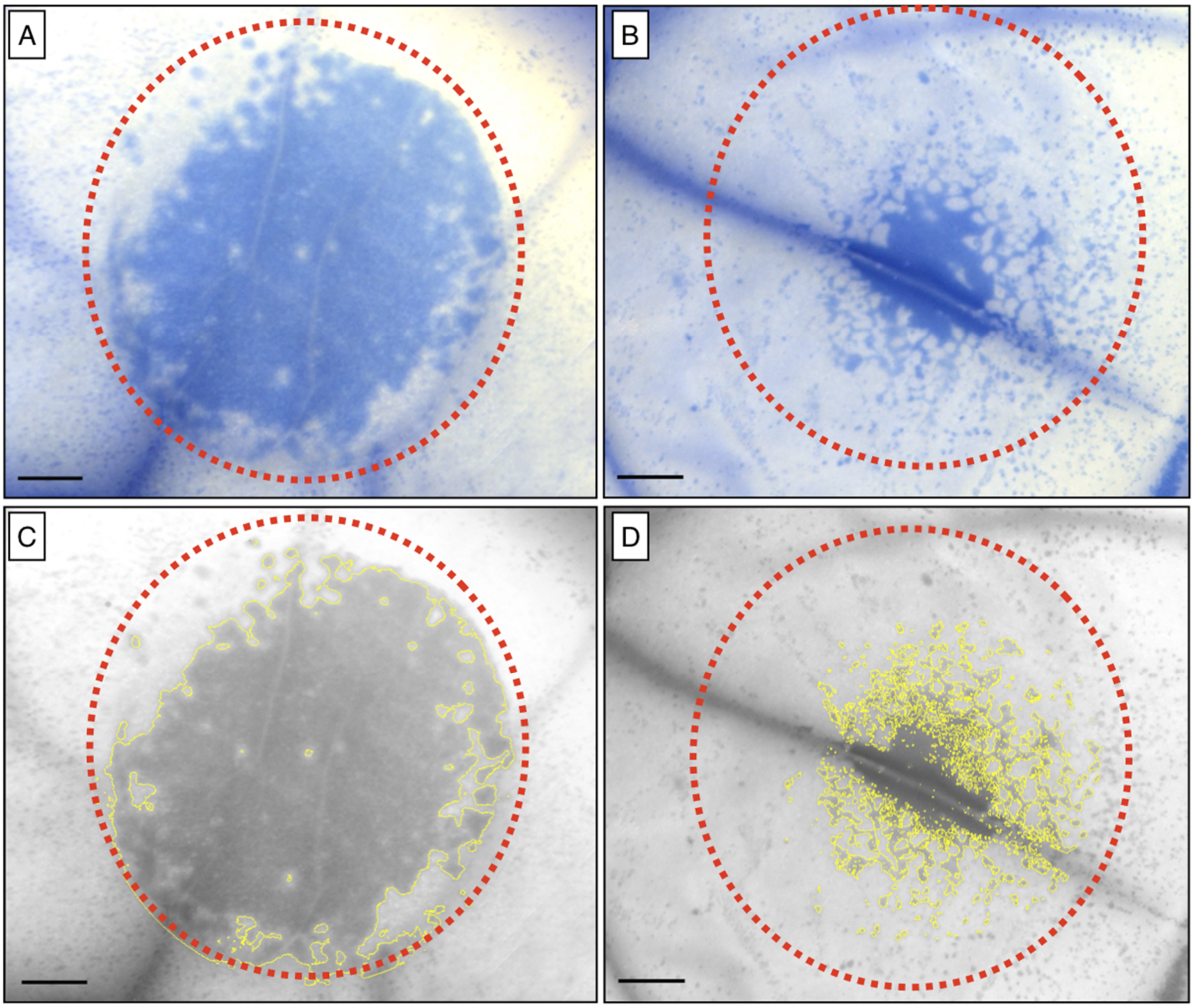

Supplementary Figure S1. Semi-automated analysis of endothelial regeneration. (**A**) and (**B**) Wounds measuring 3 mm (red dashed line) were stained with trypan blue (day 7 in culture supplemented with AR-13324). (**A**) peeled wound; (**B**) scraped wound. (**C**) & (**D**) Color Threshold analysis with Image J. The areas circumscribed by the yellow lines were detected and measured in mm^2 by Image J software. Regeneration was measured as the percentage of the area determined by the colour threshold in relation to the area encompassed by 3 mm wound. Scale bars: 500 µm.

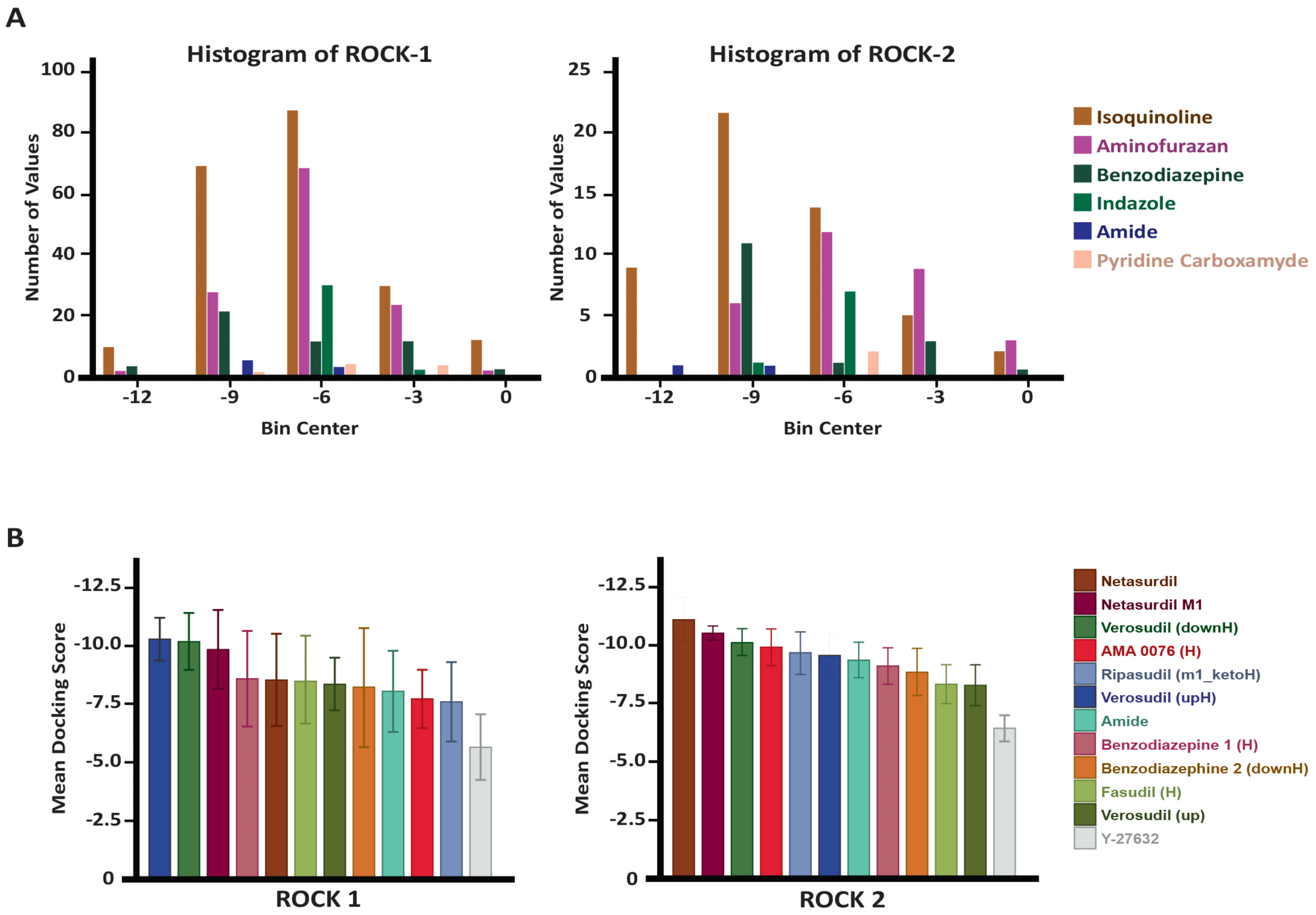

Supplementary Figure S2. *In silico* docking scores of ROCK inhibitors used in this study. Histogram showing the clustered frequency distribution of compounds from ROCK inhibitors from different classes within ranges of docking score for (**A**) ROCK-1 and ROCK-2. (**B**) Bar chart showing the mean docking scores and standard deviation of the top 10 compounds (out of 46 compounds evaluated) that had the highest docking scores for both ROCK-1 and ROCK-2 and Y-27632.

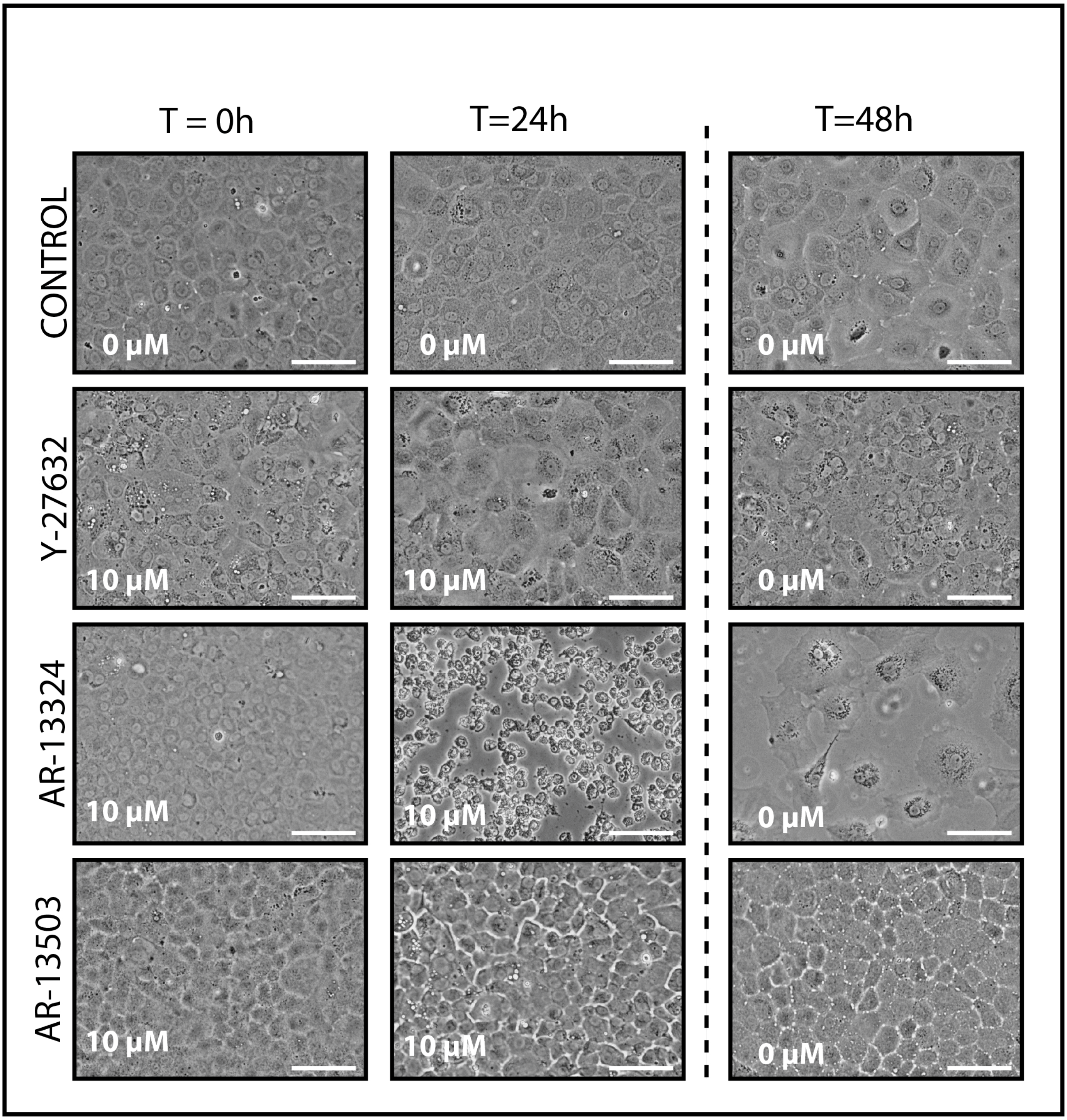

Supplementary Figure S3. Cytotoxic effects of ROCK inhibitors AR-13324 on primary CECs. Dissociated primary CECs (second passage) were seeded and left to attach to establish a monolayer (Time = 0h) before being treated with ROCK inhibitors Y-27632, AR-13324, or AR-13503 at the concentration of 10 uM for 24 hours (Time = 24h). Subsequently, ROCK inhibitors were removed with a change of fresh culture medium (depicted by the dotted line) and left for an additional 24 hours (Time = 48h) Scale bars: 100 µm.

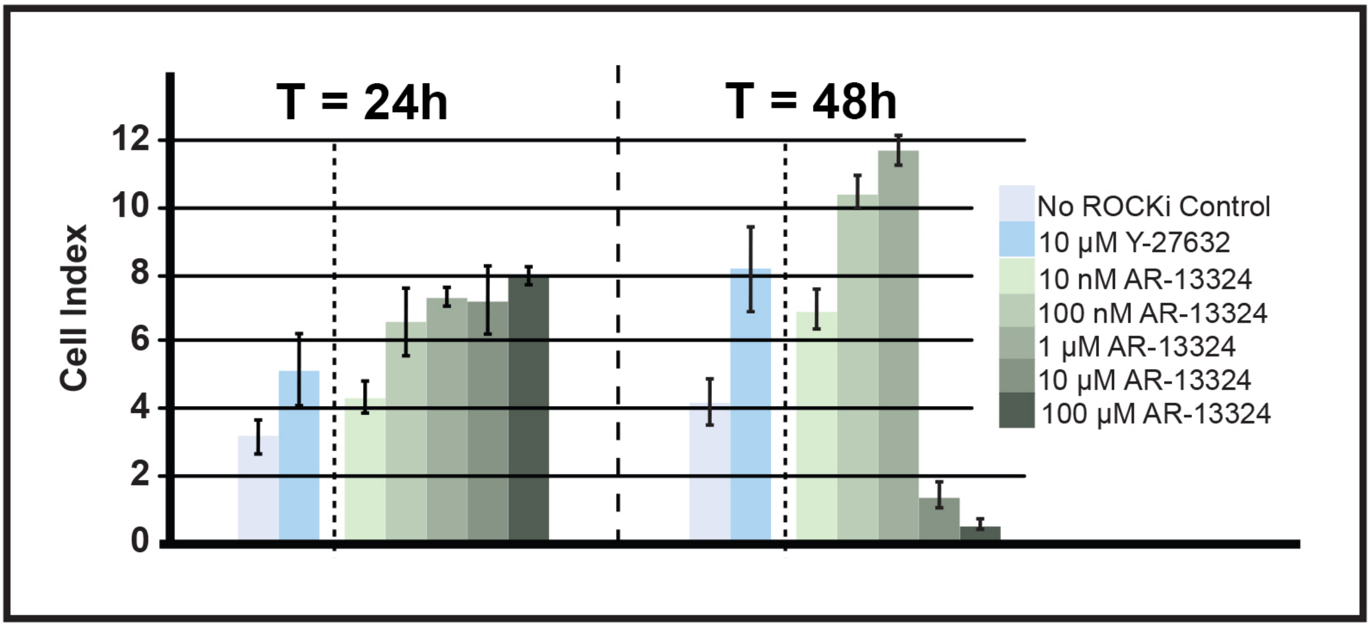

Supplementary Figure S4. Impedance readings of cellular attachment of CECs in AR-13324. Representative figure showing the effects of the extended range of ROCK inhibitors AR-13324 on primary CECs as recorded by impedance reading with the xCelligence at Time = 24h when seeded without the presence of any ROCKi (No ROCKi Control (light blue)), and in the presence of 10 µM of Y-27632 (blue), 10 nM AR-13324 (light green); and 100 nM AR-13324 (remaining 4 CECs populations) for the first 24 hours. On the following day, media were refreshed with the respective concentrations of ROCKi, and its corresponding impedance read-out were subsequently recorded following 24 hours (Time = 48h) of exposure.
